# Increase of mild disability in Japanese elders: A seven year follow-up cohort study

**DOI:** 10.1186/1471-2458-5-55

**Published:** 2005-05-30

**Authors:** Jiro Okochi

**Affiliations:** 1 Department of Health Services Coordination, Graduate School of Medical Sciences, Kyushu University, Maidashi 3-1-1, Higashi-ku, Fukuoka, 812-8582 Japan

## Abstract

**Background:**

Japan has the highest life expectancy in the world. In a 2002 census government report, 18.5% of Japanese were 65 years old and over and 7.9% were over 75 years old. In this ageing population, the increase in the number of dependent older persons, especially those with mild levels of disability, has had a significant impact on the insurance budget. This study examines the increase of mild disability and its related factors.

**Methods:**

All community-dwelling residents aged 65 and over and without functional decline (n = 1560), of Omishima town, Japan, were assessed in 1996 using a simple illustrative measure, "the Typology of the Aged with Illustrations" to establish a baseline level of function and were followed annually until 2002. The prevalence and incidence of low to severe disability, and their association with chronic conditions present at the commencement of the study, was analyzed. A polychotomous logistic regression model was constructed to estimate the association of each chronic condition with two levels of disability.

**Results:**

An increase in mild functional decline was more prevalent than severe functional decline. The accumulation of mild disability was more prominent in women. The major chronic conditions associated with mild disability were chronic arthritis and diabetes in women, and cerebrovascular accident and malignancy in men.

**Conclusion:**

This study showed a tendency for mild disability prevalence to increase in Japanese elders, and some risk factors were identified. As mild disability increasingly prevalent, these findings will help determine priorities for its prevention in Japanese elders.

## Background

Japan has the highest life expectancy in the world. In a 2002 Japanese census report, 18.5% of Japanese were 65 years old and over and 7.9% were over 75 years old. A long-term care insurance (LTCI) law was introduced in 2000 to cover both home-based and institutional care services for the large elderly population [1]. Since then, the rapid increase in the number of beneficiaries has enlarged the budgetary balance of calls and its premium rates. To access LTCI-provided services, elderly persons must comply with an eligibility test. This test is based on the physical and mental status, and it divides care needs into six categories or levels, based on the estimated amount of care resource utilization[2]. According to Ministry of Health, Labor and Welfare (MHLW) figures, beneficiaries of the at-home care service and the institutional service increased by 99% and 38%, respectively, between April 2000 and April 2003. During the same period, the number of elderly persons insured by LTCI increased by only 11%. As a result of a recent report of the MHLW, which confirmed an increase in the need for mild level care (grade 1 – support needed) from 46% to 53%, the prevention of mild disability became a focus of attention. A recent government commission on elderly care in Japan also reported that the increase in the number of elderly persons, especially those with mild disability, is endangering the insurance scheme, and the government is in the process of redesigning the scheme to refocus services for the elderly with mild disability away from direct care to preventive services. Thus, prevention or delay of the onset of functional limitation is an important objective in the health care system.

The theory of compression of morbidity suggests that life-style changes and suitable treatment for chronic illnesses can postpone the development of chronic conditions and their unwanted sequelae [3]. In the United States, later levels of disabilities and death rates are predictable from specific chronic conditions [4-7]. Very few such studies have been conducted for the Japanese population [8,9] and even fewer provide information on mild disability, which is the most common and increasing source of dependency[8].

This study aims to describe incidence and prevalence of functional decline, and to determine whether the incidence of disability at mild and severe levels is associated with age, gender and chronic conditions.

## Methods

This research combines two distinct methodologies: a longitudinal cohort study, from 1996 to 2002, of functional decline based on the entire population of the elderly in a single town, and a retrospective questionnaire study of chronic conditions in the same population.

### Longitudinal cohort study

The base population of the study was the population of Omishima town, Ehime prefecture. According to the 1995 census, the total population of the town was 4782, and elderly persons (aged over 65) numbered 1935 (40% of the population). The local municipality provided the researchers with a list of all elderly persons of 65 years and older taken from the residential register, and 1843 elderly persons living at home (95% of this total) were identified in August 1996. Persons who did not give written informed consent (n = 5) were excluded from the study, and this left an initial cohort of 1838.

### Measurement of disability

The majority of studies estimating the incidence of elderly functional decline are based on interview or questionnaire, as opposed to the probably more reliable approach of observation [10]. The present study uses an observational instrument which, because of its simplicity and ease of use, should permit more frequent observational studies of elderly functional decline. This method is the Typology of the Aged with Illustrations (TAI).

**Figure 1:**
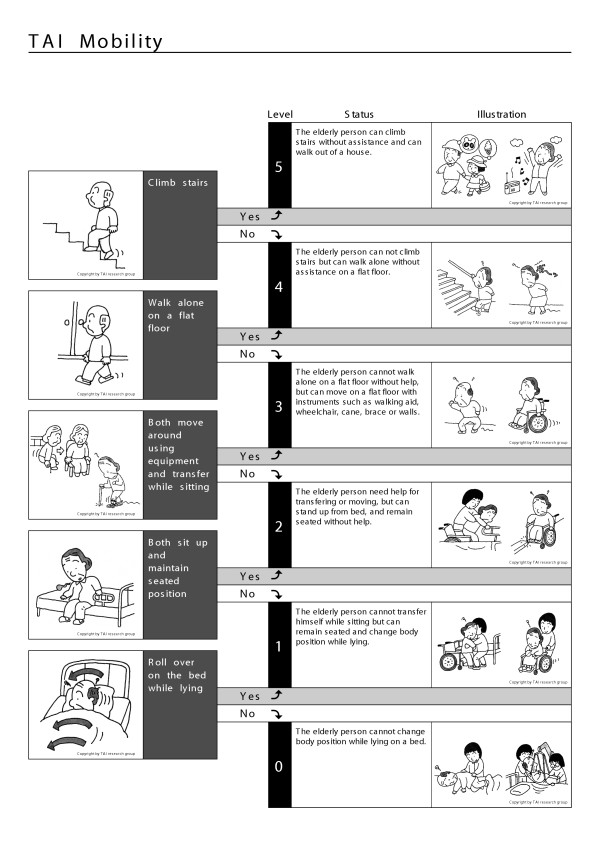
TAI mobility

**Figure 2:**
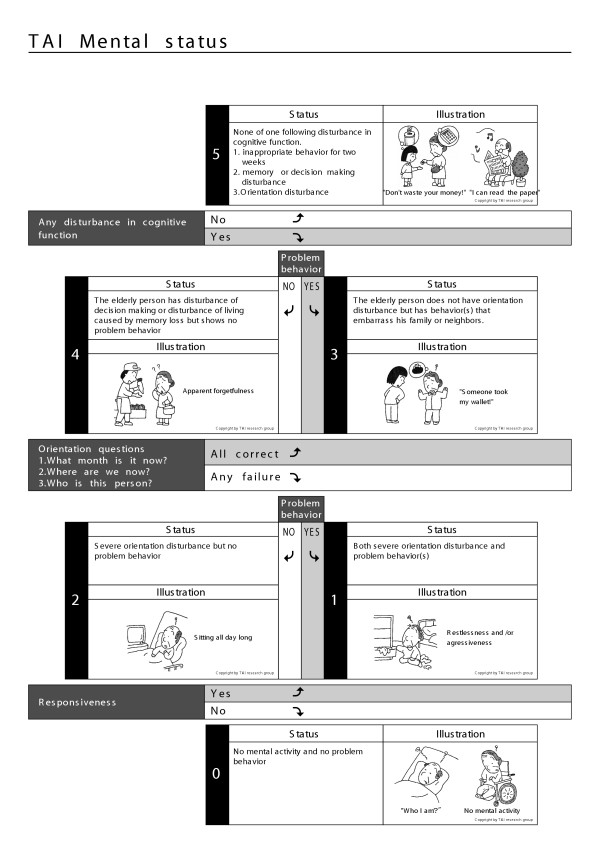
TAI mental status

**Figure 3:**
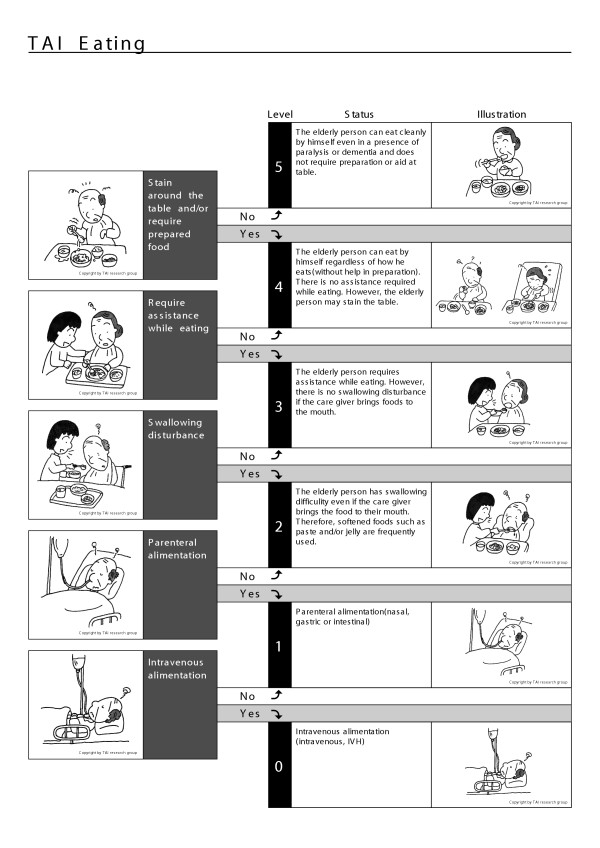
TAI eating

**Figure 4:**
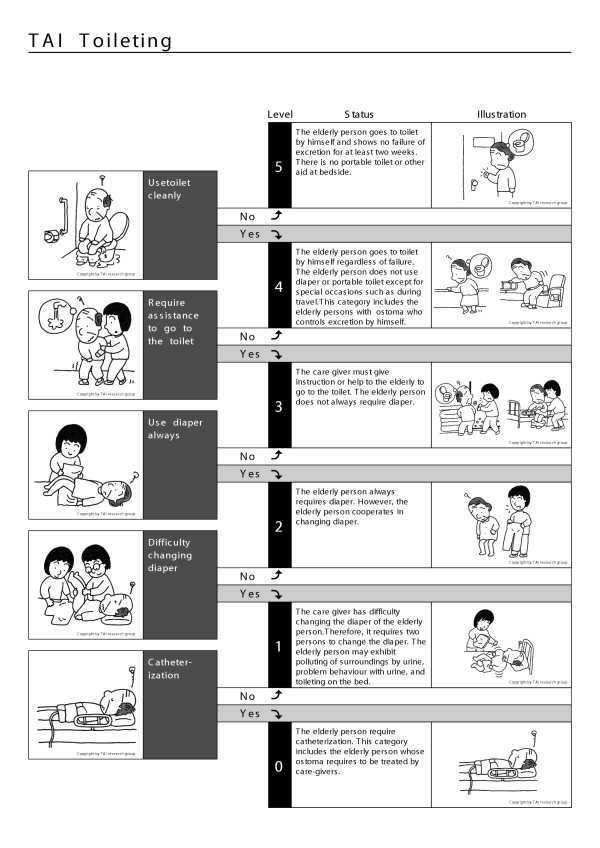
TAI toileting

The TAI is an instrument for the measurement of elderly function, and is composed of four scales representing mobility, eating, toileting, and mental status (Figures 1,2,3,4) [11,12].

Each item in the TAI has six hierarchical states (5 to 0), representing levels of disability in each domain. Five represents no disability and 0 represents extreme disability. Each state is defined by a threshold and illustrated as shown in the example of the mobility scale (Figure 1). The levels on the mobility scale are as follows: level 5, ability of the elder to climb stairs without aid or assistive devices; level 4, can not climb stairs without aid but can walk on flat surface without aid or assistive devices; level 3, cannot walk on a flat surface without aid, but can move around using assistive devices and perform transfer independently while seated; level 2, cannot either move around or transfer while seated using assistive device or aid from the others, but can sit up and maintain seated position ; level 1, cannot either sit up or maintain seated position but can roll over on the bed without aid; and level 0, cannot roll over on a bed while lying without aid.

Its reproducibility, construct validity and concurrent validity have been established in a previous study [11]. Average weighted kappa of the four scales was 0.65 and there were no significant differences between experienced and non-experienced TAI users. It has high concurrent validity with the Functional Independence Measure (FIM).

Using TAI, 1560 elderly persons were identified as not having any functional decline, and were used to assess the incidence of disability each year for successive seven years from 1996 to 2002, in order to follow its association with age, gender, living status and presence of chronic conditions. Eighteen non-professional district welfare commissioners recorded information pertinent to elderly function in the four above-mentioned domains using the TAI. Following intensive training in its use, they assessed the function of the participants in 1996, and again each August for six consecutive years. The evaluators were asked to observe and classify the present status in the measurement month using TAI mobility, eating and toileting scales. In case of TAI mental status scale, the evaluators were asked to observe and also to interview the relative functional status. In cases of cognitive impairment, the assessment was based on interviews with family members as proxies, together with observation of the elderly person.

### Retrospective questionnaire on chronic conditions

In addition to the yearly observation of elderly functions, a questionnaire covering seventeen chronic medical conditions (including the onset) was completed by the participants remaining in February 2003. Those elders who were hospitalized, institutionalized or died by 2002 were excluded from the questionnaire study since they were unable to complete the questionnaire survey. The same district welfare commissioner, who originally carried out the observation with TAI, distributed and collected the questionnaires. They also assisted respondents who had difficulty in completing the questionnaire.

The seventeen chronic medical conditions were decided based on a Ministerial statistical report on long-term care insurance law [13], and modified for the purpose of this study. They were: chronic arthritis, osteoporosis, bone fracture, chronic pain, cerebrovascular accident (CVA), heart disease, high blood pressure, diabetes, hyperlipidemia, chronic lung disease, intestinal disease, renal disease, eye disease, malignancy, depression, Alzheimer's disease and Parkinson's disease.

The questionnaire provided descriptions and definitions of the chronic conditions to facilitate understanding and eliminate recall bias as far as possible. It also included the onset years of chronic conditions. Data of social status, social activities, and health-related behavior was also recorded, but were not used in the current analysis.

### Analysis

The initial sample of 1838 was used to describe the correlation of initial disability and future severity. Of these, samples not providing a disability index due to death, emigration or institutionalization were not included in the analysis of disability index.

The data of 1560 elderly persons without initial functional decline were used to assess the incidence of disability in the population studied. Using the TAI scale, the author devised a disability index [14,15], as follows. Each of the four scales of TAI has a six-level structure (Figure 1,2,3,4). Level 5 of elderly function in each scale represents no disability and was scored as 0. At level 4, the elder has one functional problem, for example in TAI mobility, in climbing stairs, and is assigned a score of 1; at level 3, the score is 2, and so on. The results of all four scales are summed to form a single index, theoretically ranging from 0 to 20, and then divided by 20 to give each individual's score for each year of the survey.

Elders with a disability score of 0 in any year were defined as no disability. Those with scores of 0.05 to 0.10 (maximum of two disabilities) were defined as suffering from mild disabilities. Those elders with an index score equal to or greater than 0.15 (more than three disabilities) were defined as suffering from severe disabilities.

The disability-free sample (n = 1560) was used to describe the prevalence and incidence of disability. For the analysis of point prevalence, the result of each year's measurement was applied. For the analysis of incidence of new mild disability from disability-free samples, the person-year method was used. Incidence of severe disability included progression of mild disability to severe disability.

The association of disability index with gender, age and chronic medical condition, over the seven years, was analyzed using applicable data from the 1560 samples.

A polychotomous logistic regression model was constructed to test the effect of each covariate on the development of functional decline at the two levels of severity using eligible data [16]. The covariates were age at base line, living status and the seventeen chronic conditions. Only chronic conditions diagnosed before 1996 were included in the analysis so as to avoid the inclusion of acute episodes of diseases, such as CVA and bone fractures.

The associations of the chronic conditions with each of the two outcome variables were tested independently, using the chi-square or Fisher's exact test, by stratification and non-stratification of gender and endpoint functional status. Only those conditions that achieved a significance level of P < 0.05 were incorporated in the logistic regression.

Finally, as the population studied showed gender differences in functional decline, separate models for men and women were constructed. All p values were two-tailed. The analyses were conducted using SPSS 11.5.1J, Windows.

## Results

The cohort of 1838 elderly persons aged 65 and over was 40% male at the beginning of the study in 1996. Age range was 65 to 99 years, and average age was 73.6 years (SD 6.5) for males and 74.8 years (SD 7.1) for females.

When first observed in 1996, 1560 (85 %) of these elders had no functional decline, 180 (10 %) showed a mild level of disability, and 98(5%) showed severe disability. Additional file 1 shows the change of the status from 1996 to 2002. Higher transition to severe disability was more prominent in mild disability group (14%) compared with no disability group (4%). There was a difference of transition from no disability to mild disability between genders (male 10% versus female 23%). The transition from no disability to dead was higher in male (26% versus 13%).

The average age of sample without disability of men (n = 654) and women was 73.0(SD6.0) and 73.6(SD6.3), respectively.

By 2002, 289 of the original 1560 participants without initial disability had died, 53 were lost to follow up or emigrated, and 50 were hospitalized or institutionalized. All of them were excluded from the analysis of risk factors. Of the 1168 participants remaining in the study in 2002, 1107(96%) participants provided the initial living status data and were measured for all consecutive 7 years, and 1067 (93%) responded to the questionnaire in 2003.

**Figure 5:**
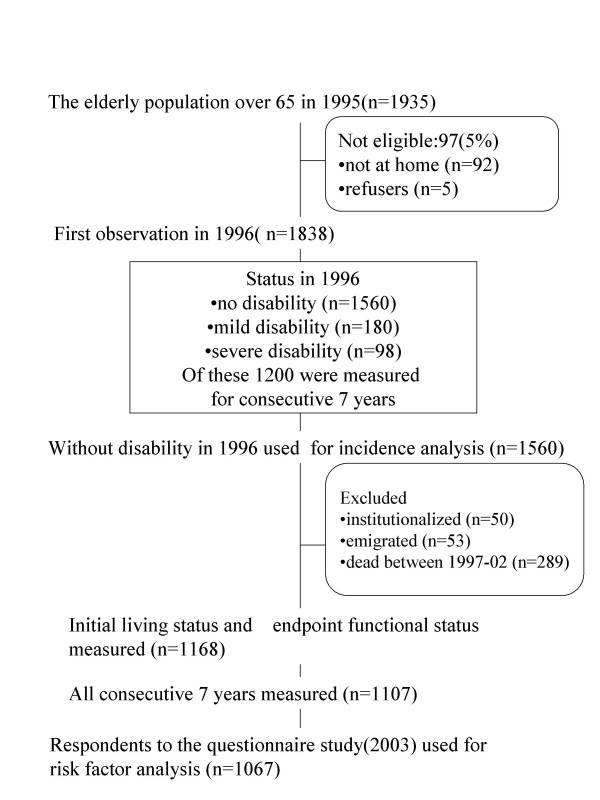
Population of elderly people living at home: Flow of subjects through the study

Figure 5 summarizes the subjects' progress through the study.

The disability index in 1996 was significantly higher in those who died before the last measurement in 2002 using the 1273 surviving cases and 433 deaths (Mann-Whitney's U Test, P < 0.001). These 1200 cases who were measured for consecutive 7 years were analyzed to show the result of the rank correlation between the initial disability index and the disability index of subsequent years, after excluding elderly who died (n = 433), emigrated (n = 66), or were unable to participate further due to hospitalization or institutionalization (n = 66) (Table 1).

**Table 1:** The Mean and median of disability index and rank correlation between disability index of 1996

	year	1996	1997	1998	1999	2000	2001	2002
Male (n = 453)	Mean	0.006	0.006	0.010	0.012	0.015	0.018	0.027
	SD	0.035	0.034	0.053	0.058	0.060	0.067	0.080
	Median	0.003	0.003	0.003	0.004	0.007	0.008	0.012
	Correlation*		0.629	0.475	0.433	0.363	0.324	0.327
	P		<0.001	<0.001	<0.001	<0.001	<0.001	<0.001

Female (n = 747)	Mean	0.007	0.009	0.009	0.011	0.016	0.022	0.036
	SD	0.031	0.032	0.035	0.043	0.055	0.061	0.082
	Median	0.005	0.006	0.006	0.007	0.010	0.014	0.020
	Correlation*		0.615	0.521	0.460	0.363	0.324	0.256
	P		<0.001	<0.001	<0.001	<0.001	<0.001	<0.001

*Spearman's rank correlation between disability index of 1996

Table 2 shows the distribution of disabilities, according to TAI grade, of subjects at the two levels of the index of disability, in1996. For example, of those with mild disability (n = 180), 141 (78%) could not climb stairs by themselves, but could walk on a flat surface without aid or assistive devices, and 13 (7%) could move about only on a flat surface with aid. Forty-three (24%) had mild memory problems and 15 (8%) had mild difficulty using toilet. Only two had a problem with eating. In those with mild disability, 145 (81%) showed disability on only one scale, while 35 (19%) showed disability on two scales. 84 percent of the elders had disability only in the mobility scale and this suggested that the mild disability group is composed mostly of the elders with mobility problem, without other functional problem.

**Table 2:** Disability level and result measured with the typology of the aged with illustrations (TAI) in 1996

mild disability (n = 180)				
**TAI level**	mobility	mental	eating	toileting

5	14%	76%	99%	92%
4	78%	24%	1%	8%
3	7%	1%	1%	0%
2	0%	0%	0%	0%
1	0%	0%	0%	0%
0	0%	0%	0%	0%

severe disability (n = 98)				

**TAI level**	mobility	mental	eating	toileting

5	4%	44%	61%	16%
4	21%	27%	28%	34%
3	32%	1%	7%	20%
2	21%	21%	2%	16%
1	15%	3%	1%	12%
0	6%	4%	1%	1%

Subjects with severe disability (n = 98) had a variety of functional impairment. In this group, only two cases (2%) showed disability on only one scale, both of which involved mental status dysfunction, and 4 cases (4%) had no problem with mobility.

Only one subject had a TAI mental level of 3 and only four a TAI mental level of 1, all of whom exhibited problem behaviors, as shown in the Figure 2.

### Prevalence and incidence of disability

**Figure 6:**
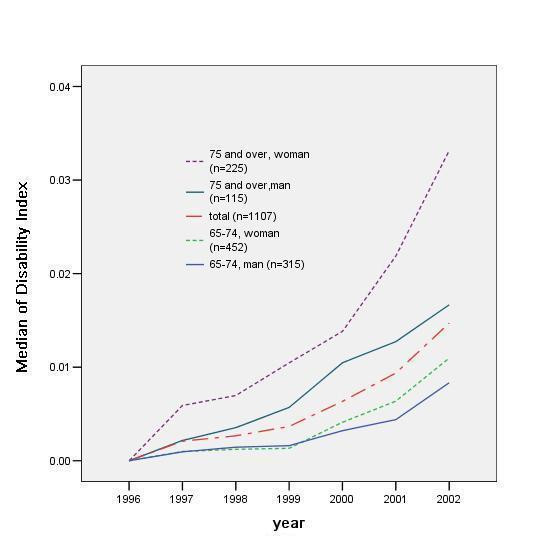
Pattern of disability index median, in gender and age groups (n = 1107)

Figure 6 shows the change of the median of the disability index for consecutive seven years (n = 1107). In this analysis, only the samples that were measured for consecutive 7 years were included, and therefore, the elderly persons who were hospitalized, institutionalized, dead or emigrated were excluded. Older age at base line had an effect on the rate of the disability development. In base line age group older than 75, the increase of the disability index was more prominent in woman than that of man after year 2000. Most of the curves, except for the man aged 75 and over, showed an exponential increase pattern.

The associations of gender and age with scores on the disability index were tested separately on yearly data using the eligible samples from the same population (n = 1107). Gender difference was not obvious from 1997 to 2001, but in 2002 women showed a higher mean disability index score than men (male 0.22, female 0.30, T test, P < 0.05). Age at enrolment, in 1996, correlated positively with disability index scores for every year of measurement (Spearman's rank correlation, P < 0.01).

**Figure 7:**
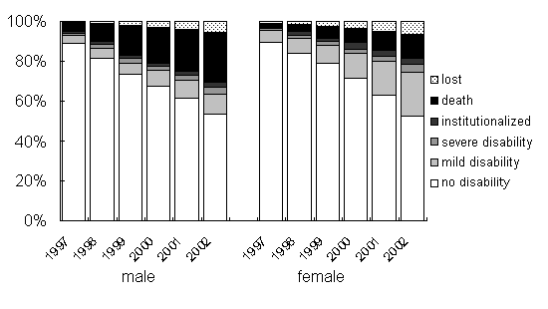
Prevalence of disability, institutionalization and death in men versus women without initial disability (n = 1560)

The point prevalence of disability at the two severity levels, and of institutionalization and death, is shown in Fig. 7. Mild disability was more increased in women than in men, rising to 22% in women versus 10% in men by 2002. By contrast, loss from the study population by death was more common in men, reaching 26% in men versus 13% in women by 2002. The proportion of severe disability in 2002 was 3.4% and 4.2% for men and women, respectively, and the proportion of elders who were institutionalized in 2002 was 3.4% and 4.2%, respectively.

As shown in Table 3, age-group in 1996 also had an effect on the development of the disability. In men, higher age group showed higher proportion of death in 2002, while institutionalization was higher in younger age group. In women, both the proportion of death and institutionalization were higher in older age group.

**Table 3:** Age and gender difference of the disability, institutionalization and death without initial disability (n = 1560)

**gender**	age group		no disability	mild disability	severe disability	institution*	emigrated†	death
**Male**	65 to 74	n	271	38	14	15	11	78
	(n = 427)	%	63	9	3	4	3	18
	75 and over	n	82	29	8	4	10	94
	(n = 227)	%	36	13	4	2	4	41
	Total	n	353	67	22	19	21	172
	(n = 654)	%	54	10	3	3	3	26

**Female**	65 to 74	n	375	94	12	15	11	39
	(n = 546)	%	69	17	2	3	2	7
	75 and over	n	108	111	26	16	21	78
	(n = 360)	%	30	31	7	4	6	22
	Total	n	483	205	38	31	32	117
	(n = 906)	%	53	23	4	3	4	13

* including hospitalization

† including loss from the sample with unknown reasons

**Figure 8:**
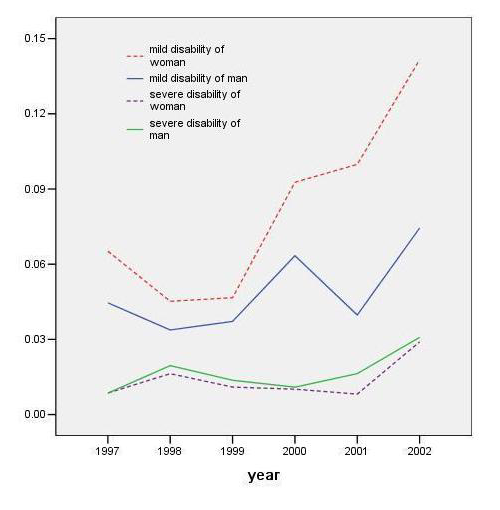
Incidence of disability by severity and gender

Figure 8 shows the yearly incidence of new cases at the two levels of disability by gender. A high incidence of mild disability compared to severe disability was particular to females. The incidence of death was higher in men in all 6 consecutive years (data not shown).

### Factors associated with disability

Additional file 2 shows baseline chronic conditions in 1996, cross-tabulated with the outcome level of disability by gender. The average age of males (n = 405) completing this part of the study was 71.6 years (SD 4.9), and of females, 72.6 years (SD 5.8). At least one chronic conditions was reported by 671 (61.4%) of this group.

The association of number of chronic conditions with scores on the disability index was tested separately on yearly data using this sample (n = 1067). The number of chronic condition was correlated with disability index score in 1998 (P < 0.05), 2000, 2001 and 2002 (P < 0.01, respectively).

The selection of elderly persons without functional decline at the commencement of the study excluded the participants suffered from Alzheimer disease or other dementia. Depression (n = 11), Parkinson's disease (n = 7) and Alzheimer disease (n = 0), which were too low in prevalence in 1996 to permit statistically meaningful analysis, are not included in this table. Chronic arthritis, osteoporosis, bone fracture, cerebrovascular accident (CVA), diabetes, chronic lung disease, eye disease and malignancy all showed significant associations with level of disability (chi-square test or Fisher's exact test, stratifying and non-stratifying the outcome severity level).

The chronic conditions with statistically significant associations and age at initial measurement were used to construct the polychotomous logistic regression model shown in Table 4. The conditions found to be related to mild disability in males were CVA and malignancy, and that to severe disability was CVA. The chronic conditions related to mild disability in women were chronic arthritis and diabetes, and those related to severe disability were chronic arthritis and CVA. The results for severe disability must be interpreted cautiously, because of the limited number in the end-point sample; the confidence interval for relative risk is larger than that for mild disability.

**Table 4:** Associations of Chronic Conditions and Age with Functional Decline in Participants Without Initial Functional Limitation (n = 1067)

		outcome level					
		mild			severe		
Gender	Covariate	R.R	95% C.I.	P	R.R	95% C.I.	P
Male							
	age*	2.5	(1.4–4.5)	P < 0.01	5.3	(2.1–13.2)	P < 0.01
	chronic arthritis	1.9	(0.6–6.2)		1.6	(0.3–8.6)	
	osteoporosis†	4.4	(0.5–36.2)		n.a		
	bone fracture	2.6	(0.8–8.1)		1.7	(0.2–14.8)	
	CVA§	5.6	(1.7–19.1)	P < 0.01	20.3	(5.2–78.6)	P < 0.01
	diabetes	1.2	(0.4–3.1)		2.5	(0.6–10.1)	
	chronic lung disease	2.2	(0.9–5.6)		2.9	(0.7–12.0)	
	eye disease	1.0	(0.4–2.4)		1.0	(0.3–3.6)	
	malignancy†	5.4	(1.6–18.3)	P < 0.01	n.a		

Female							
	age*	4.9	(3.4–7.1)	P < 0.01	9.0	(4.4–18.2)	P < 0.01
	chronic arthritis	2.8	(1.5–5.2)	P < 0.01	5.4	(1.9–15.8)	P < 0.01
	osteoporosis	1.4	(0.6–3.2)		2.6	(0.7–9.9)	
	bone fracture	1.3	(0.6–3.1)		1.1	(0.3–4.9)	
	CVA§	3.4	(0.6–19.8)		22.3	(2.5–198.5)	P < 0.01
	diabetes	2.6	(1.2–5.9)	P < 0.05	1.5	(0.2–13.2)	
	chronic lung disease	0.3	(0.1–1.1)		1.5	(0.3–8.0)	
	eye disease	1.1	(0.6–1.8)		0.8	(0.3–2.3)	
	malignancy†	0.8	(0.3–2.3)		n.a		

*effect of ten-year increase, † insufficient numbers in category, §cerebrovascular accident

Because of the relatively low prevalence of chronic conditions, the sum of chronic conditions suffered was used to determine the effect of multiple conditions. The relative risks, of adding 1 chronic condition for severe and for mild disability were 1.2 (95%CI 1.0–1.4) and 1.2 (1.1–1.3), respectively, controlling for age and gender.

## Discussion

The aim of the current study was to describe incidence and prevalence of disability and to identify the effect of a age, gender, living condition and chronic conditions as risk factors of functional decline in Japanese elders, and to associate them with different degrees of disability. The identification of risk factors that correlate with the development of mild disability, and which serve as are suitable targets for prevention, is of particular importance in today's society, where increasing prevalence of mild disability and of costly dependency of the aged is clearly apparent [17,18]. The present study is also of interest for its use of a base population in which the proportion of elders, aged 65 and over was 40%. To the best of the author's knowledge, this is the most aged society studied epidemiologically to date.

### Prevalence and incidence of disability

This study initially used the disability index to show the occurrence of disability in the population. This index had an exponential distribution, i.e. the most of the elders have no disability as shown in Table 1 and Figure 6, since the sample represents a normal population, as is in a previous study[14].

The speed of disability development was different among age-groups and genders, suggesting that there is different underlying process for developing disability among these groups (Figure 6). To examine this difference, the author divided the disability into two categories; mild and severe disability. And the change was prominent in the mild disability group (Figure 7).

Although the mortality rate of this cohort was within the range of that of other studies, the prevalence of overall disability was higher than in some other studies in Japan [19-21]. One study, for example, reported a lower rate of mobility disability compared to the present study [21]. This may be because of fine categorization in TAI definitions, as it classifies the elders who have problem to climb stairs into mild disability. In a previous study, stair climbing was categorized to be the difficult task, compared to other ADL and mobility items[22]. The measurement instrument in this study employs it as a tool to detect mild disability. Repeated measurement will likely show a higher chance of identifying more disability[23], and the very aged population might also have been responsible for this difference in prevalence.

The present study found an increase of mild disability in the cohort, especially in women. These findings appear to differ from those of previous studies which found that men show a faster decline than women in the Japanese population[21]. However, this result is in accordance with that of women having a longer survival time, and therefore the disability accumulates in women [24]. In woman, the transition from no disability to mild disability was higher in both age groups than men (Table 3). Higher disability index after 2000 in woman aged 75 and over also supports the accumulation of disability in woman. In men, higher age group showed higher proportion of death in 2002, but it did not apply to the cases of institutionalization. This suggested the non-exponential pattern of increase of disability index median in men (Figure 6) was attributable to the death, but not to institutionalization.

The gender difference of the proportion of elders with severe disability was not as prominent as with mild disability. These results suggest different factors are associated with the development of disability in two genders, especially in the development of mild functional limitation.

### Factors associated with disability

Earlier studies in Japan have identified a variety of chronic conditions as related to the development of task specific ADL or IADL disability [19,25,26]. The association of chronic diseases with both physical and cognitive function has been investigated [8,19]. However, to date, no studies using a cohort design and a Japanese sample have reported the association of number and type of chronic conditions with severity levels of disability to the best of the authors' knowledge.

It is reasonable to hypothesize that different kinds of chronic conditions will have different functional sequelae, and there is some empirical evidence both in the US and in Japan that different risk factors are associated with reduced performance on different levels of disability.

Previous non-Japanese studies have estimated the risk associated with chronic conditions for the development of different levels of functional or ADL disability[4,6,7]. In the present study, the principle associates of both levels of disability for men were CVA and malignancy, while in females they were chronic arthritis, CVA and diabetes, as shown in Table 4. These findings are similar, but not identical, to those found in a previous Japanese study [25].

Some chronic conditions might relate to earlier death of the participants. The weak association between the number of chronic conditions and the disability index in earlier years, namely 1997 and 1999, might be due to exclusion of deceased and institutionalized cases.

Of the chronic condition studied, CVA is the most frequently cited as to have association with functional decline [8,25,27], but it has been shown that, because of the short survival time after stroke, the number of dependent elderly persons does not necessarily increase as a result [21]. This study also showed the association of the severe disability and CVA. And the incidence and prevalence of disability did not increase as much as the mild disability.

In contrast, chronic arthritis is consistently found to be a risk factor for both genders, and shows no association with mortality [4]. As might be expected, studies have indicated that the prevention of disabilities consequent on non-fatal conditions, such as chronic arthritis, is the most cost-effective preventative strategy [17,18]. The present study confirmed the significance of chronic arthritis, in women only, both for its high prevalence (9%) and its high relative risk for the development of both mild and severe disability.

This study also found the association between diabetes and mild disability in woman. In a Japanese population, Kishimoto et al. reported that, a history of diabetes is associated with poor performance on more ADL tasks in women than in men [26]. Diabetes has been shown to be associated with slower walking speed, inferior lower extremity function, and decreased balance[28], all of which meet characteristics of mild disability in the present study.

Many previous studies have suggested bone fracture and osteoporosis are risk factors for functional disability [29,30]. In the present study, however, while bone fracture and osteoporosis, in women only, appeared to be associated using Fisher's exact test, it failed to show a relationship in the logistic regression model. Ross et al. have suggested that the risk of falls among Japanese women is lower than for Caucasian women [31]. The low prevalence of these conditions in non-disabled persons may have contributed to this result. In addition, it is possible that the six-year analysis interval used in the current logistic regression analysis was too long for the detection of effects of bone fracture[23].

The prevalence of the chronic condition that achieved statistical significance with chi-square test was highest in eye disease in women, but it did not show association in the logistic regression model. Next to it was the chronic arthritis, osteoporosis and bone fracture, followed by the diabetes. In men, chronic lung disease is the highest followed by the CVA then chronic arthritis. This result suggested different approach in prophylaxis is required to prevent accumulation of disability in the population.

### Study limitation

The present study has a few limitations. The history of physician-diagnosed chronic medical conditions and self-reports of the same were obtained retrospectively. A previous study had found that self-report of chronic conditions in the elderly was accurate [32], but inaccurate recall of the time of onset of chronic conditions was present, especially for arthritis [33]. Current ignorance of the prevalence of chronic conditions among well-functioning Japanese elders also limits the interpretation of the prevalence of chronic conditions among this sample. The exclusion from the analysis of participants who died or were institutionalized or emigrated in the course of the study, some of whom may have exhibited a chronic condition at baseline, may also have affected the results since those who were included for the analysis of risk factors were younger and thus were presumably healthier. The absence of information regarding to the levels of severity of the chronic conditions reported, and the relatively low prevalence of each chronic condition, meant that the associations measured were less specific than could be desired. Chronic conditions such as osteoporosis and Parkinson's disease that did not achieve statistical significance in this study may in fact contribute to the development of disability with a larger sample. Some conditions could be related to the development of disabilities in shorter or longer period of observation.

This study did not incorporate those elders who were institutionalized or dead at the endpoint for the analysis of the risk factors. This is because only 24 percent of the institutionalized cases provided responses to the questionnaire study, and none did so in the deceased cases, compared to 90 percent of the surviving cases. Inclusion of these endpoints could have improved association with the risk factors.

In addition, caution should be exercised with regard to extrapolation of the results to other populations due to the use of a single base population. However, the present study does have the advantage of using a whole population rather than a sample. By using geographically defined area, this study had little loss of the data throughout the 7 consecutive years.

Other methodological approach of analysis, such as the use of Structural Equation Model (SEM) could have been more appropriate with this data. However the stability of the model when applied for this analysis was poor, mainly because of the distribution of the endpoint variables used in this study.

Despite the limitations, this study is significant in that it provides information on the incidence and prevalence in Japan of two levels of disability – mild and severe – and gives indication of priorities in the selection of chronic conditions for prophylaxis, especially as regards to the elderly with mild disability over a lengthy period. In the context of long-term care insurance in Japan, and plans to direct services for mildly impaired elderly persons towards rehabilitation, this study can be employed to develop suitable objectives in the prevention of unwanted sequelae of chronic conditions. This study also suggested that man and woman require different prophylaxis, because different factors were associated with the development of disabilities in two genders.

Population-based studies using TAI in another Japanese town, at two- and six-month observation intervals have been initiated by the author and collaborators, in order better to understand the functional loss process and its risk factors.

## Conclusion

This study showed a tendency for mild disability prevalence to increase in Japanese elders, especially in women. This study also identified some risk factors in the development of mild disability; chronic arthritis and diabetes for women and the CVA for men. In Japan, the budgetary balance of the newly instituted long-term care insurance system is endangered by increase in the mildly impaired elderly, and these findings should help determine priorities for prevention.

## Competing interests

The author has not received reimbursements, fees, funding, or salary from an organization that may in any way gain or lose financially from the publication of this manuscript, either now or in the future.

The authors does not hold any stocks or shares in an organization that may in any way gain or lose financially from the publication of this manuscript, either now or in the future. I am not applying for any patents relating to the content of the manuscript. The author of this article has not received reimbursements, fees, funding, or salary from an organization that holds or has applied for patents relating to the content of the manuscript. I do not have any other financial competing interests.

## Authors' contributions

Jiro Okochi carried out the study design, data collection, statistical analysis and preparation of the manuscript.

## Pre-publication history

The pre-publication history for this paper can be accessed here:



## Supplementary Material

Additional File 1 The status change of the participants between 1996 and 2002 by gender (n = 1838) Click here for file

Additional File 2 Baseline characteristics, prevalence of chronic conditions and endpoint functional status of remaining participants in 2002 (n = 1067) Click here for file
